# Fatty acid oxidation and photoreceptor metabolic needs

**DOI:** 10.1194/jlr.TR120000618

**Published:** 2021-02-06

**Authors:** Zhongjie Fu, Timothy S. Kern, Ann Hellström, Lois E.H. Smith

**Affiliations:** 1 Department of Ophthalmology, Boston Children’s Hospital, Harvard Medical School, Boston, MA, USA; 2 Manton Center for Orphan Disease, Boston Children’s Hospital, Boston, MA, USA; 3 Center for Translational Vision Research, Gavin Herbert Eye Institute, Irvine, CA, USA; 4 Section for Ophthalmology, Department of Clinical Neuroscience, Institute of Neuroscience and Physiology, Sahlgrenska Academy, University of Gothenburg, Göteborg, Sweden

**Keywords:** lipid metabolism, mitochondrial fuel, retina, CPT, carnitine palmitoyltransferase, 3HB, D-3-hydroxybutyrate, Nrf2, nuclear factor E2-related factor 2, OXPHOS, oxidative phosphorylation, RPE, retinal pigment epithelium, TCA cycle, tricarboxylic acid cycle, VLDLR, VLDL receptor

## Abstract

Photoreceptors have high energy demands and a high density of mitochondria that produce ATP through oxidative phosphorylation (OXPHOS) of fuel substrates. Although glucose is the major fuel for CNS brain neurons, in photoreceptors (also CNS), most glucose is not metabolized through OXPHOS but is instead metabolized into lactate by aerobic glycolysis. The major fuel sources for photoreceptor mitochondria remained unclear for almost six decades. Similar to other tissues (like heart and skeletal muscle) with high metabolic rates, photoreceptors were recently found to metabolize fatty acids (palmitate) through OXPHOS. Disruption of lipid entry into photoreceptors leads to extracellular lipid accumulation, suppressed glucose transporter expression, and a duel lipid/glucose fuel shortage. Modulation of lipid metabolism helps restore photoreceptor function. However, further elucidation of the types of lipids used as retinal energy sources, the metabolic interaction with other fuel pathways, as well as the cross-talk among retinal cells to provide energy to photoreceptors is not fully understood. In this review, we will focus on the current understanding of photoreceptor energy demand and sources, and potential future investigations of photoreceptor metabolism.

## Photoreceptor fuel sources

**Fig. 1:**
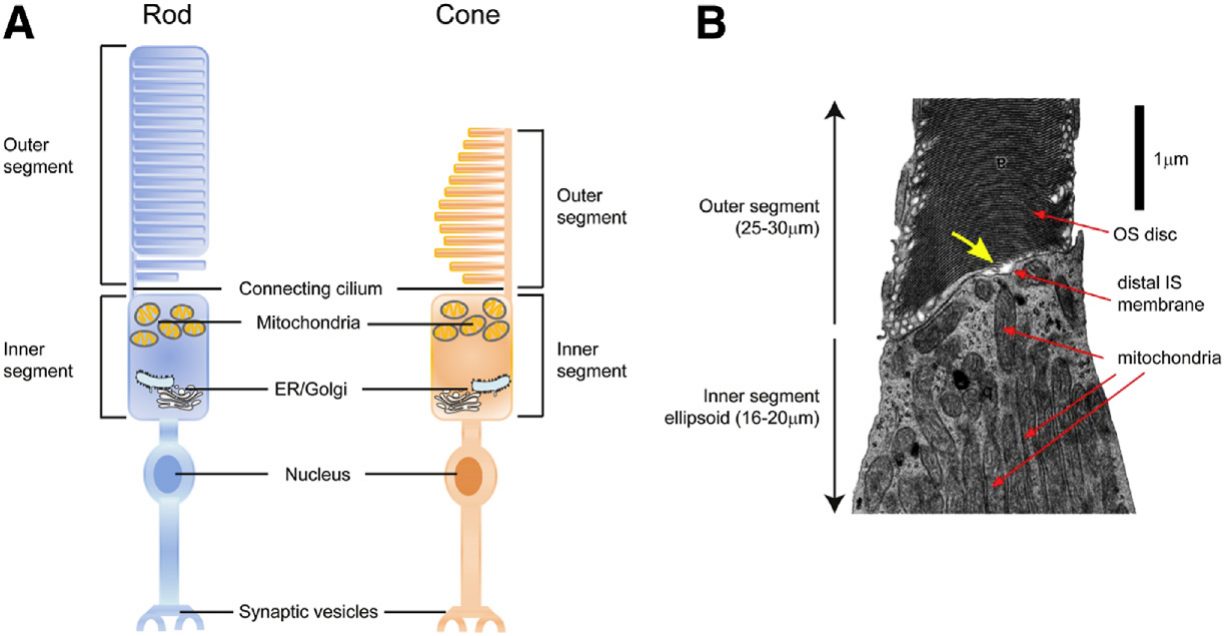
Schematics of rod and cone photoreceptor structure. A: Photoreceptor consists of outer segment, inner segment, nucleus, and synaptic vesicles. Inner segment (IS) consists of mitochondria-rich ellipsoid and ER/Golgi complex-rich myloid. Outer segment (OS) is rich in lipids and undergoes shedding daily. B: An electron micrograph of a partial human cone photoreceptor [reprinted with permission from (150)]. IS ellipsoid is densely packed with mitochondria. In OS, the stacked discs are clearly visible. A narrow gap is visible between the distal IS membrane and proximal discs of the OS (yellow arrow). If this gap is an artifact of tissue preparation for electron microscopy or whether it corresponds to a region of interstitial fluid that separates the IS and OS is unknown.

Light responsive retinal rod and cone photoreceptors (Fig. 1) require high energy production for maintenance of the “dark current,” for phototransduction and for the 10% daily replacement of shed photoreceptor outer segments (1, 2, 3). During phototransduction, photons are converted into electrical impulses. In the light, sodium (Na^+^) ion channels close to hyperpolarize photoreceptor membranes, leading to the suppression of glutamate release and photoreceptor excitation. In the dark, more than half of photoreceptor energy is used to maintain a steady flow of Na^+^ into the cell (the dark current), allowing cellular depolarization and glutamate release, and thus inhibition of photoreceptor excitation (4, 5, 6). Photoreceptors also shed distal outer segments daily (rich in lipids vulnerable to damage from light and oxidation). Continuous shedding of “used” outer segment discs and replacement with newly assembled discs is critical to maintain normal photoreceptor function (7). The inner segments of rods and cones has been identified as a site of phospholipid synthesis in frogs injected with radioactive glycerol (8). Both retinal pigment epithelium (RPE) and Müller glial cells are involved in the support of outer segment renewal (9, 10, 11, 12), although retinal lipid processing in these cells is not yet fully understood.

**Fig. 2:**
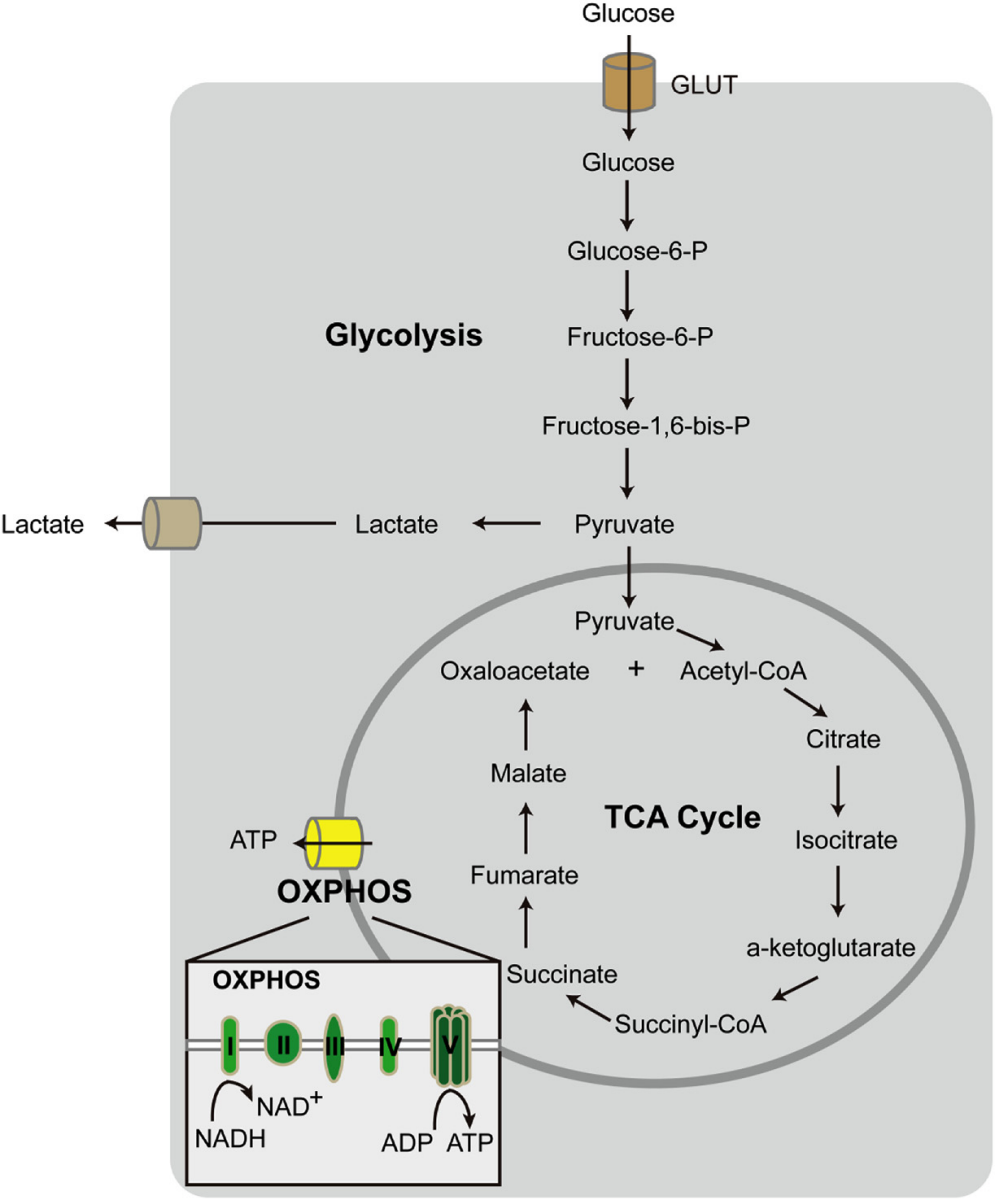
Cellular metabolism of glucose. Glucose enters cells through glucose transporter (GLUT) and is converted to pyruvate. In the absence of oxygen, pyruvate is converted to lactate and the process is known as glycolysis. In the presence of oxygen, pyruvate enters mitochondria and undergoes TCA cycle. At the mitochondrial membrane, reduced electron carriers, NADH (and FADH2), from the TCA cycle pass their electrons to protein complexes (I to V) for ATP production, a process known as OXPHOS.

Although retinas are known to be a high energy-demanding tissue, with a very high density of mitochondria, retinal fuel sources are not yet clearly delineated. We know however that aerobic glycolysis of glucose is important. In the retina and tumors, glucose is mostly metabolized into lactate by glycolysis rather than through oxidative phosphorylation (OXPHOS) for energy production (13, 14, 15, 16). In addition, glucose can also be converted to carbon dioxide, glutamate, γ aminobutyrate, aspartate, glutamine, and traces of alanine in the retina (17). It is important to note that both aerobic glycolysis and respiration are approximately doubled in mature versus immature rat or rabbit retinas, possibly because of increased differentiation and better intercellular connection of the visual cells (17). (Fig. 2). Photoreceptors express high levels of hexokinase II and pyruvate kinase M2, as well as lactate dehydrogenase subunit A, which favor the conversion of pyruvate to lactate (18, 19, 20, 21, 22). Glycolytic intermediates and the regulation of glycolytic rate are crucial for outer segment biosynthesis (21). Only 20% of glucose is oxidized and 80% is used for glycolysis in pig retinal explants (13) so there must be alternate fuel sources for OXPHOS in the retina. Photoreceptors are capable of taking up and metabolizing lactate when glucose is scarce (23, 24, 25). Dystrophic versus normal rat retinas show a marked depression in incorporation of [^14^C]glycine into total protein before differentiation of the tissue has begun, both in vivo and in vitro (26). The pentose phosphate pathway, a metabolic pathway parallel to glycolysis, is highly active in dystrophic retinas throughout early development and progression of the lesion (26). Recently palmitate (C16:0) has been shown to be a fuel substrate for mitochondrial energy production (27). Knowledge of the types of fatty acids that have impact on photoreceptor metabolism is still very limited.

## Retinal lipid composition

Photosensitive discs in rod outer segments consist of proteins and lipids that are primarily phospholipids (90–95%) and cholesterols (4–6%) (7, 28). The composition of the phospholipid fatty acid chains in human retina is not fully defined, but in the retina of healthy seniors there are five major types of fatty acids: DHA (22:6n3, 15.3%), AA (20:4n6, 11%), palmitic acid (16:0, 20.8%), stearic acid (18:0, 20.1%), and oleic acid (18:1n9, 13.8%) (29). DHA and AA are essential lipids that cannot be synthesized in sufficient quantities by mammals and are mainly acquired from the diet. DHA is found primarily in oily cold-water fish such as salmon, sardines, and mackerel, while AA is present in animal products (meat and egg yolk) (30). The retina achieves preferential accretion of DHA by three mechanisms: *1*) conserving and recycling of phospholipid-esterified DHA in the shed rod outer segment disc via phagocytosis by RPE (31, 32, 33, 34), *2*) uptake of dietary preformed DHA from the circulation (34, 35, 36), and *3*) a small component from synthesis of DHA from α-linolenic acid (18:3n3, dietary precursor of DHA) by RPE (37, 38). Although the retina expresses elongases and Δ5/Δ6 desaturases necessary for biosynthesis of α-linolenic acid to DHA, the rate of synthesis is low (39), and may be inadequate to support the optimal DHA level in photoreceptors. DHA is essential for the maintenance of photoreceptor function and morphology, as well as the inhibition of pathological retinal angiogenesis (40, 41, 42). Supplemental DHA is associated with a lower risk of proliferative retinopathy of prematurity (43). AA is also an essential fatty acid and low circulating levels of AA are associated with a higher risk of developing vision-threatening retinopathy of prematurity in extremely premature infants (44).

Although studies of other fatty acids (palmitic acid, stearic acid, and oleic acid) in retinal energy metabolism and function are still lacking, we may gain some fundamental understanding from their impact on other tissues. Palmitic acid can be oxidized to carbon dioxide (CO_2_) in animal tissue with high endogenous respiration (45). Human fetal tissues (brain, lung, and liver) metabolize palmitic acid to lipids and CO_2_ in vitro (46). These findings suggest that palmitic acid can be a fuel substrate for mitochondrial energy production in some tissues. In mouse retinas ex vivo, palmitic acid directly increases the oxygen consumption rate, which reflects mitochondrial respiration (27). In rats with constant LED light exposure, light-induced oxidative stress causes reduction in retinal DHA levels, which in turn affects the stearic acid composition and increases membrane rigidity (47, 48). In general, decreased membrane rigidity leads to increased cell metabolism and also higher division rates (49). For example, in mammary carcinoma, tumor-bearing rats in vivo, stearic acid supplementation reduces the ratio of stearic to oleic acid in erythrocyte membranes (49). Inhibition of stearoyl-CoA desaturase, which catalyzes the conversion of stearic acid to oleic acid, leads to tumor growth delay in a human gastric cancer xenograft model (50). Oleic acid plays a role in the balance and transport of retinoids and fatty acids in the retina, as it binds more strongly than does DHA to bovine interphotoreceptor retinoid-binding protein (51). In gastric cancer and breast cancer cells in vitro, oleic acid activates AMP-activated protein kinase and enhances mitochondrial energy production (52). However, our knowledge of stearic and oleic acid in cell function is mainly from a membrane property perspective, and their impact on retinal metabolism needs to be explored.

## Lipid as photoreceptor energy source

Palmitate has been shown to be a mitochondrial fuel substrate in ex vivo mouse retinas from mice deficient in VLDL receptor (VLDLR) (27). VLDLR is found abundantly in organs (heart, skeletal muscle) with high metabolic rates (53). In heart and skeletal muscle, fatty acids are oxidized to acetyl-CoA, which enters the Krebs cycle for energy production in mitochondria (53, 54). In the presence of oxygen, the complete oxidation of one glucose molecule yields 30–32 ATP molecules, whereas one palmitate molecule yields 106 ATP molecules. In the eye, VLDLR is expressed in RPE and photoreceptors (27, 55, 56). VLDLR facilitates lipid entry into the cell by anchoring apolipoprotein E-triglyceride-rich lipoproteins and enables lipoprotein lipase to cleave long-chain fatty acids from triglycerides (57, 58). VLDLR deficiency leads to decreased uptake of fatty acids and fatty acyl intermediates of β oxidation (27). Palmitate supplementation increases oxygen consumption in wild-type but not VLDLR-deficient mouse retinas ex vivo; blocking carnitine palmitoyltransferase (CPT) (to block fatty acid entry into mitochondria) with etomoxir prevents palmitate-induced retinal oxygen consumption (27). These observations suggest that retinas can use lipids as direct mitochondrial fuel substrates. Moreover, proteins involved in fatty acid β oxidation have been reported in RPE, photoreceptors, and Müller glial cells (59, 60, 61, 62). Genetic mutations in enzymes involved in fatty acid oxidation pathways, such as trifunctional enzyme subunit α (HADHA), trifunctional enzyme subunit β (HADHB), or long-chain 3-hydroxyacyl-CoA dehydrogenase, cause trifunctional protein deficiency, and mitochondrial deficiency, leading to pigmentary retinopathies and vision loss (63, 64). PPARα (a nuclear receptor) modulates lipoprotein lipase expression and triglyceride metabolism (65). Loss of PPARα in mice causes decreased lipid metabolism and neurodegeneration (66). Activation of PPARα with fenofibrate reduces the progression of diabetic retinopathy by 30–40% as seen in two large-scale clinical trials (FIELD and ACCORD Eye studies) (67, 68). In rodent models of pathological retinal angiogenesis, fenofibrate reduces retinal neovascularization and retinal vascular leakage (69, 70). Fenofibrate may also exert retinal neurovascular protective effects and modulate lipid metabolism as a CYP2C antagonist (71, 72). CYP2C metabolites from ω-3 and ω-6 long-chain polyunsaturated acids are pro-angiogenic in murine models of proliferative retinopathies (73, 74). Taken together, defects in fatty acid β oxidation pathways may lead to energy deficiency in the eye and cause retinal dysfunction. Further study is needed to elucidate the modulation of retinal lipid use and its role in retinal metabolic disorders.

### RPE and photoreceptors

RPE plays a vital role in maintaining photoreceptor homeostasis and RPE metabolism is tightly linked to that of photoreceptors. Cell culture models suggest that lactate suppresses glucose consumption and enhances transport of glucose across a monolayer of human RPE cells in vitro (75), supporting the hypothesis that RPE preferentially passes glucose to the photoreceptors (76). Enhancing RPE glycolysis causes death in neighboring photoreceptors in mice (77). In addition to the transport of glucose to photoreceptors, circulating radioactive [^3^H]palmitate and stearic acid taken up by RPE are concentrated in oil droplets and in the cytoplasm of the RPE, and then appear to be taken up by photoreceptor outer segment membranes, with only minimal amounts taken up in the inner segment that have high concentrations of mitochondria where fatty acid β oxidation occurs (78). However, more recent studies suggest that RPE cells are able to transfer fatty acids to photoreceptors.

The RPE produces retinyl ester-containing lipid droplets (retinosomes), replenishing the visual chromophore 11-*cis*-retinal to ensure proper visual function (79, 80). Lipid storage is dysregulated in some retinal degenerative diseases such as age-related macular degeneration (81, 82). In *Drosophila* and mouse retinas, overexpression of RPE-specific fatty acid transport protein induces lipid droplet accumulation and increased energy metabolism in RPE and retinas (83). Vesicles with comparable size to lipid droplets are transferred from RPE to photoreceptors and found in close association with photoreceptor mitochondria in *Drosophila* retinas (83). These observations suggest that RPE-derived lipid droplets may potentially provide energy substrates for photoreceptors. Interestingly, in diverse vertebrate species (avian and Xenopus), there are also local oil droplets (consisting of neutral lipids and carotenoids) found within the inner segment of cone photoreceptors. In chicken photoreceptors, neutral lipids in oil droplets are composed of cholesterols, mono-, di-, and triacylglycerols specifically enriched for polyunsaturated fatty acids, including linoleic acid and AA (84). These oil droplets may modify the intensity and spectrum of light reaching the photosensitive outer segment, but their function is not fully understood. Further investigations into the role of local lipid droplets in photoreceptor energy metabolism are needed.

Ketone body metabolism is a significant contributor to overall energy metabolism within extrahepatic tissues in mammals (85). Ketone bodies are primarily produced in the liver from fatty acid oxidation-derived acetyl-CoA. Circulating ketone body levels increase after prolonged exercise or fasting (85). Ketone bodies D-3-hydroxybutyrate (3HB), acetoacetate, and acetone serve as energy sources during nutritional deprivation (86). Primary human fetal RPE cells in vitro highly express mitochondrial HMG-CoA synthase 2 (HMGCS2), the rate-limiting enzyme in ketogenesis, and metabolize exogenous palmitate to produce 3HB (87). RPE phagocytoses photoreceptor outer segments to produce 3HB (88). RPE can take up and metabolize ^13^C-labeled 3HB into tricarboxylic acid cycle (TCA cycle) intermediates and amino acids (87). In an optic nerve and central retinal blood vessel transection model, exogenous 3HB protects against ischemic retinal degeneration with enhanced antioxidative defense through upregulating nuclear factor E2-related factor 2 (Nrf2) and Nrf2 activator fumarate (86). Ketogenesis plays a key role in supporting RPE metabolism, preventing lipid accumulation and protecting retinal neurons.

### Müller glial cells and photoreceptors

Müller glial cells span the entire retina and are in direct contact with photoreceptors, with blood vessels and with many other retinal neurons. The metabolic impact of these cells is not fully understood. Many studies (some seemingly contradictory) have used in vitro Müller glial cells. Primary Müller glial cells isolated from guinea pigs produce lactate which is shuttled to photoreceptors for conversion to pyruvate to fuel OXPHOS (24). Cultured primary human retinal Müller glial cells obtain ATP principally from glycolysis and have a low rate of oxygen consumption in the presence of glucose and oxygen (89). Conversely, primary Müller glial cells isolated from mice are deficient in pyruvate kinase (22). These cells produce little lactate in culture but metabolize lactate and aspartate produced by photoreceptors (22). Enzymatic histochemistry in mouse retina also shows that Müller glia may rely on the TCA cycle to generate GTP and P transferring kinases to produce ATP to support glial cell energy requirements (90). Cultured human Müller glial cells (MIO-M1) highly express glutamate transporters and increase glutamate uptake in response to glucose deprivation (91). Rat Müller glial cells (TR-MUL) are able to convert glutamate to α-ketoglutarate in vitro (92).

Glial cells promote synapse formation. In cultured retinal ganglion cells, the frequency and amplitude of spontaneous postsynaptic currents increases dramatically after coculturing with glial cells (93, 94). This process might be ApoE-dependent. ApoE is a component of circulating plasma lipoproteins and involved in local transport of cholesterol and other lipid transport processes. Cholesterol is required for neuronal function, glia-dependent synapse formation, and visual signaling because a high-cholesterol diet substantially corrects the retinal abnormalities in a mouse model of Smith-Lemli-Opitz syndrome with hereditary defects in cholesterol biosynthesis (95). Cultured Müller glia from adult white rabbit synthesize ApoE, and the secreted ApoE is efficiently assembled into lipoprotein particles (96, 97). There is increased ApoE immunoreactivity in Müller glial cells from degenerative human retina (98), in line with increased ApoE in response to brain insults (99). ApoE is primarily produced in astrocytes and microglia in brain (100). Loss of ApoE in mice is associated with synaptic loss and neurodegeneration (101). These observations suggest that Müller glial cell-derived lipoproteins might be a potential source of lipids for photoreceptors for synapse formation and energy production.

As noted, most Müller glial cell metabolic studies are in vitro. It is difficult to extrapolate results from in vitro studies that have loss of normal cell interaction between Müller glial cells and their connecting neurons to Müller glial cell metabolism in vivo. In addition, primary Müller glial cells in vitro are positive for GFAP, which reflects Müller glial cell gliosis under stressed conditions (102, 103). Therefore, the metabolic properties of Müller glia and their relationship to photoreceptors in vivo need to be evaluated.

**Fig. 3:**
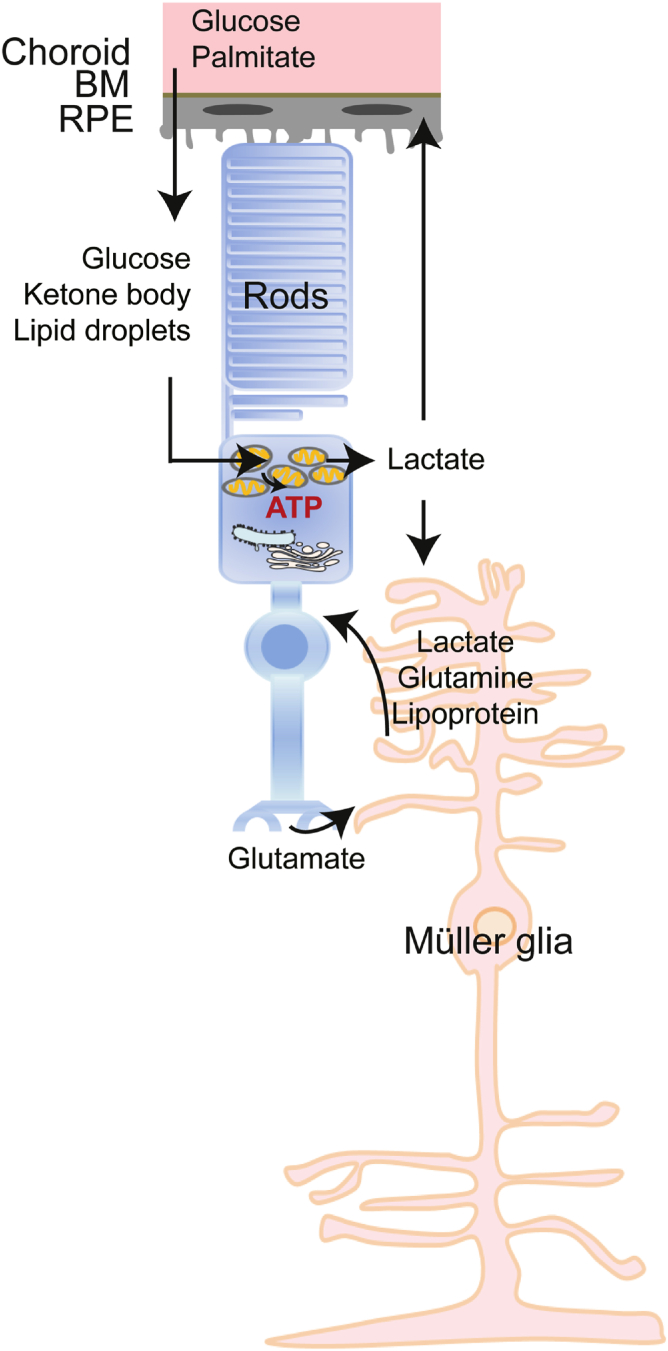
Schematic of potential metabolic links among photoreceptors and support cells. RPE cells may uptake: *1*) glucose from the choroid and transport it to photoreceptors where glucose is converted to lactate through glycolysis and then used by RPE as metabolic fuel; *2*) palmitate from the choroid and convert it to ketone bodies, which are then used in photoreceptors as energy fuel. RPE may also transfer lipid droplets to photoreceptors for energy production. Photoreceptors may spare lactate to fuel Müller glial cells. Müller glial cells uptake glutamate released from photoreceptors and convert it to glutamine. Glutamine as well as lactate and lipoprotein from Müller glial cells may be transported to photoreceptors and used as energy substrates. The metabolic links need to be further validated. BM, Bruch’s membrane.

An overall metabolic link among photoreceptors and their supporting cells (RPE and Müller glia) is summarized (Fig. 3). However, further validation is needed and exploration of other potential energy substrates is ongoing.

## Lipid hemostasis

### Peroxisomal and mitochondrial β oxidation

**Fig. 4:**
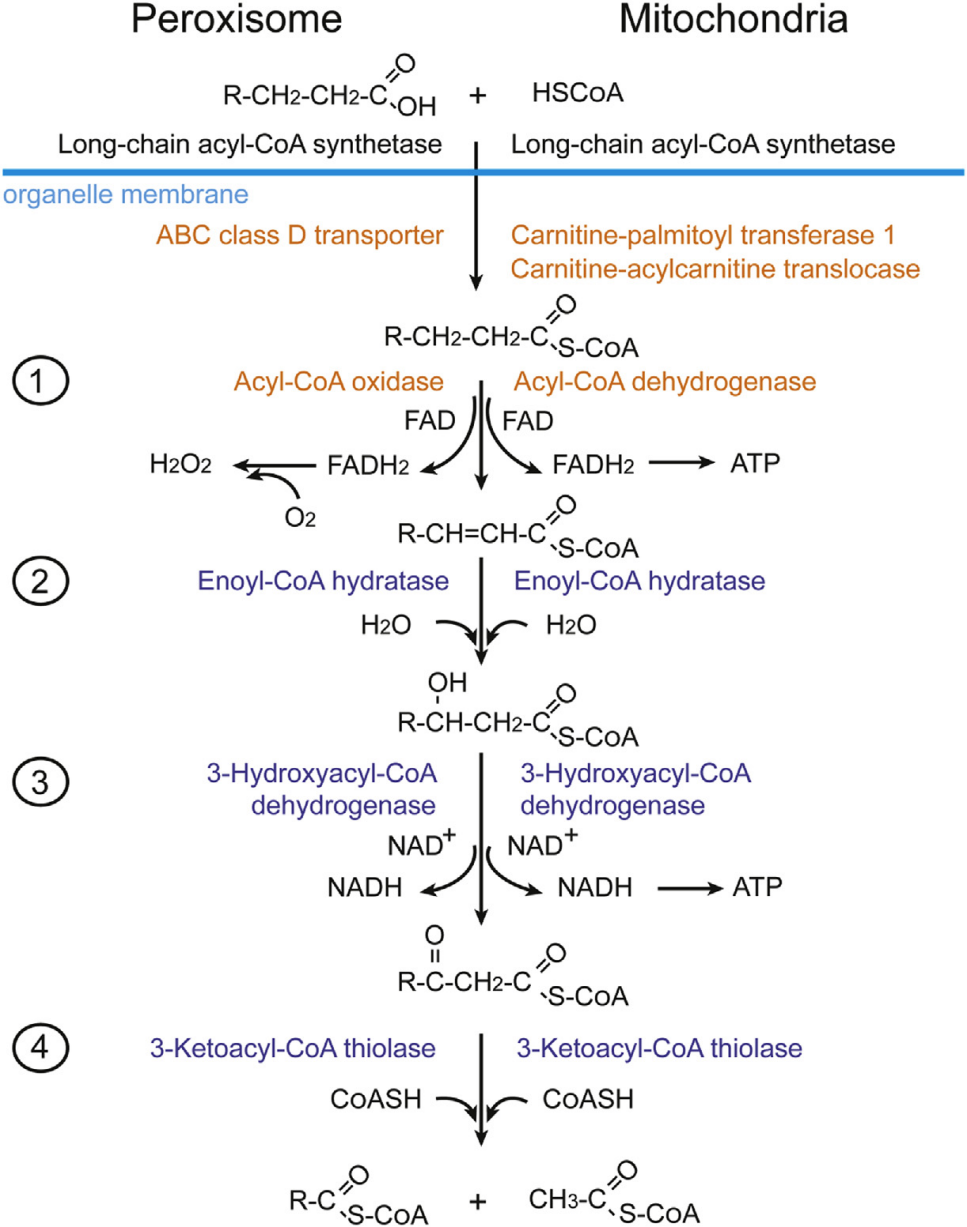
Beta oxidation of fatty acids in peroxisomes and mitochondria. To enter the organelle, long-chain fatty acids conjugate to either CoA (peroxisomes) or carnitine (mitochondria); and then the conjugated fatty acids are imported into organelles by ABC class D transporters (peroxisomes) or carnitine-acylcarnitine translocases (mitochondria). In the organelle, fatty acids go through four consecutive reactions and are converted to acetyl-CoA and acyl-CoA. Acetyl-CoA later enters the TCA cycle for ATP production.

Fatty acid breakdown through fatty acid β oxidation occurs in peroxisomes (without ATP production) and in mitochondria (with ATP production) in mammals (Fig. 4). Peroxisomal degradation oxidizes very long-chain monocarboxylic (≥22 carbons) and long-chain dicarboxylic fatty acids (104). Oxidation of polyunsaturated fatty acids occurs faster in peroxisomes than in mitochondria (105). In order to enter peroxisomes or mitochondria, long-chain fatty acids (13–21 carbons) first need to conjugate to either CoA (peroxisomes) or carnitine (mitochondria) outside the organelle. The conjugated fatty acids are then imported into organelles by ABC class D transporters (peroxisomes) or carnitine-acylcarnitine translocases (mitochondria). β oxidation degrades fatty acids into acetyl-CoA, which enters the Krebs cycle and is oxidized into CO_2_ and water. In addition, FADH_2_ and NADH are also released after β oxidation and Krebs cycle processing, and then are used for energy production by the mitochondrial electron transport chain.

### Anti-oxidant system

Mitochondria’s highly oxidizing microenvironment is the largest source of ROS, including superoxide anion, hydroxyl radical, hydrogen peroxide, and singlet oxygen (106). For example, the superoxide anion and hydroxyl free radicals have an unpaired electron, which, in excess, can damage cell membranes and lipoproteins (107). Hydrogen peroxide in excess can also penetrate cell membranes and induce cell death (106). To deal with excessive ROS, cells build antioxidant enzyme defenses, including superoxide dismutase, glutathione, and catalase (only in peroxisomes) (108). Oxidative stress is caused by an imbalance between the production of ROS and the antioxidant defense system and influences the development and progression of multiple retinal disorders including retinopathy of prematurity, age-related macular degeneration, glaucoma, diabetic retinopathy, and retinal vein occlusion. Knockdown of superoxide dismutase in RPE causes retinal dysfunction and degeneration of the RPE, as well as the shortening and disorganization of the photoreceptor outer and inner segments (109). During aging, catalase activity, but not superoxide dismutase activity, decreases in both macular and peripheral RPE isolated from human donors (110). In diabetic rats, retinal superoxide dismutase and catalase activity decreases by 40% and 32%, respectively (111). Glutathione, which degrades hydrogen peroxide, is found in photoreceptor outer segments, Müller glial cells, and retinal horizontal cells, as well as in RPE (112). Lutein, which is characterized by its blue light-filtering and anti-oxidant properties, reduces retinal oxidative stress and exerts neuroprotective effects in ischemic retinopathies (113, 114).

To modulate the anti-oxidant system and maintain cellular homeostasis, the Nrf2 protects against oxidative stress in photoreceptors and RPE (115, 116). Nrf2 is a master antioxidant transcription factor, inducing the expression of genes encoding enzymes involved in detoxication, anti-oxidant production, carbohydrate metabolism and NADPH regeneration, as well as lipid metabolism (β-oxidation and lipases) (117). Adeno-associated virus-mediated delivery of Nrf2 promotes retinal neuronal survival in mouse models of photoreceptor degeneration and acute nerve damage (118, 119). Targeting Nrf2 and its downstream antioxidant factors may possibly benefit neurons in degenerating retinas.

### Autophagy

Autophagy is a lysosome-dependent cellular degradation process that scavenges cellular components in response to environmental and cellular stress such as nutrient starvation, infection, or excess ROS (120). Autophagy processes protein aggregates, dysfunctional organelles, intracellular pathogens, and storage nutrients (glycogen and lipid droplets), thereby maintaining cellular homeostasis. Autophagy provides sources of energy and recycles building blocks for the synthesis of macromolecules (120). In the retina, autophagy proteins, including autophagy-related protein 9 (*Atg9*) and microtubule-associated protein 1A/1B light chain 3 (*LC3*), are strongly expressed in cell layers with high metabolic demand and a propensity for mitochondrial damage, such as the ganglion cell layer, a subpopulation of cells in the inner and outer nuclear layer (photoreceptors) as well as the RPE (121, 122). In photoreceptor inner segments (rich in mitochondria), autophagy is associated with lipofuscin granule accumulation (123). Basal autophagy is essential to maintain rod integrity and phototransduction (124); the lack of autophagy is associated with retinal degeneration in autophagy gene 5 (ATG5)-deficient mice. In aging RPE, seen with age-related macular degeneration, there is an accumulation of proteins and damaged cell organelles (125, 126), suggesting a changed autophagy flux. However, it is not clear whether alterations in autophagy are the cause or a consequence of retinal diseases.

## Balance between lipid use and other energy substrates in retinas

### Lipids and glucose

To match nutrient availability, tissue (including retina) may adapt fuel utilization to improve metabolic efficiency (14). For example, many cancer cells primarily metabolize glucose through aerobic glycolysis for energy production even when adequate oxygen is available for OXPHOS (the Warburg effect). Meanwhile, they also use amino acids (glutamine, serine, branched-chain amino acids) and fatty acids for cellular maintenance and energy production (127, 128, 129, 130). In gliomas, the most common form of malignant brain tumor, glycolysis supports energy production and provides carbon skeletons for the synthesis of nucleic acids, while fatty acids are utilized as energy substrates and as raw materials for lipid membranes. However, in hepatocytes or adipocytes, malonyl-CoA from glycolysis inhibits CPT1 (facilitating long-chain fatty acid transport from the cytosol into mitochondria) (131, 132), sparing lipids from fatty acid oxidation for storage in lipid droplets. In the retina with VLDLR deficiency, poor lipid uptake and resulting high circulating lipids (through free fatty acid receptor 1) suppress retinal glucose transporter 1 (GLUT1) expression, which decreases glucose uptake (27). However, other control mechanisms for fuel substrate selection and the interaction between glucose and lipid metabolism in retinal cells are unknown.

### Lipid and amino acids

Although we have some knowledge regarding glucose and lipid metabolism in the retina, there is little published concerning retinal lipid and amino acid metabolic interaction. In macular telangiectasia type 2 (MacTel), a rare macular disease that leads to central vision loss, low serine levels may cause elevated levels of toxic deoxysphingolipids, increasing the risk for disease progression (133, 134). In highly metabolic cells like the human colon cancer cell line HCT116, serine deprivation compromises mitochondrial ceramide metabolism and inhibits cell proliferation (135). Further elucidation of the role of serine and sphingolipid metabolism in retinal neurovascular function is needed. In addition to serine, other amino acids like arginine and glutamine also potentially contribute to retinal disease development. In patients with proliferative diabetic retinopathy versus nondiabetic controls, vitreous arginine and acylcarnitine levels are increased (136). Plasma glutamine and glutamic acid levels (and their ratio) might be novel biomarkers for developing diabetic retinopathy in type 2 diabetes (137). Systemic administration of arginine-glutamine dipeptide inhibits retinal neovascularization in mouse oxygen-induced retinopathy, modeling some proliferative aspects of diabetic retinopathy (138). Although glutamine and lipid metabolic interaction studies are very limited in the retina, glutamine influences lipid metabolism in other tissues. In response to hypoxia, neurons increase fatty acid biosynthesis from glutamine/glutamate in vitro (139). In isolated rat hepatocytes in vitro, glutamine stimulates lipogenesis and reinforces glucose-dependent decrease in ketone-body production (reflecting inhibition of fatty acid β oxidation) (140). The interaction between retinal glutamine and lipid metabolism also needs to be further explored.

## Fatty acid metabolism in other retinal cell types

In addition to photoreceptors, the impact of mitochondrial fatty acid metabolism on other retinal cells has also been investigated. RPE cells in vitro metabolize palmitate to produce β-hydroxybutyrate, a potential substrate for photoreceptors to maintain retinal cell health and function (87, 141). Loss of mitochondrial fatty acid transporter CPT1a in retinal endothelial cells in vivo causes impaired cell proliferation, but not impaired migration, by decreasing de novo nucleotide synthesis for DNA replication (142). However, reduction of fatty acid oxidation in retinal endothelial cells does not lead to energy depletion or disturb redox homeostasis (142). M2 phenotype macrophages (with a higher angiogenic potential than M1 in vitro) contribute to pathological retinal angiogenesis in vivo (143, 144). Shifts in cellular metabolism seem to play a role in macrophage function and phenotype (145). M1 macrophages rely mainly on glycolysis while M2 macrophages are more dependent on OXPHOS. Amino acid-sensing machineries are required for M2 polarization in vitro (146), and fatty acid oxidation is not essential for M2 polarization, suggested by myeloid cell-specific knockdown of CPT2 in mice (147, 148). Further investigation of fatty acid oxidation and the metabolic cross-talk in various retinal cells is required to better understand the lipid metabolic impact on retinal neurovascular function.

## Conclusions

Our current knowledge of photoreceptor fuel sources is limited. Recent reports have shown that fatty acid oxidation plays a role in maintaining photoreceptor metabolic homeostasis. As dyslipidemia has been reported in retinal disorders (149), targeting the restoration of normal lipid metabolism, and understanding the interaction of lipid with other mitochondrial fuel substrates in all retinal cells may help retinal disease prevention and treatment.

## Conflict of interest

The authors declare that they have no conflicts of interest with the contents of this article.
